# Neonicotinoid-induced pathogen susceptibility is mitigated by *Lactobacillus plantarum* immune stimulation in a *Drosophila melanogaster* model

**DOI:** 10.1038/s41598-017-02806-w

**Published:** 2017-06-02

**Authors:** Brendan A. Daisley, Mark Trinder, Tim W. McDowell, Hylke Welle, Josh S. Dube, Sohrab N. Ali, Hon S. Leong, Mark W. Sumarah, Gregor Reid

**Affiliations:** 1 Canadian R&D Centre for Human Microbiome and Probiotic Research, Lawson Health Research Institute, London, N6A 4V2 Ontario Canada; 2 Department of Microbiology and Immunology, The University of Western Ontario, London, N6A 5C1 Canada; 3 London Research and Development Center, Agriculture and Agri-Food Canada, London, N5V 3V3 Canada; 4 Vrije Universiteit Amsterdam, Faculty Earth and Life Sciences, Institute of Molecular Cell Biology, Amsterdam, 1081 Netherlands; 5 Department of Surgery, Division of Urology, University of Ottawa, Ottawa, K1Y 4E9 Canada; 6 Department of Surgery, The University of Western Ontario, London, N6A 4V2 Canada

## Abstract

Pesticides are used extensively in food production to maximize crop yields. However, neonicotinoid insecticides exert unintentional toxicity to honey bees (*Apis mellifera*) that may partially be associated with massive population declines referred to as colony collapse disorder. We hypothesized that imidacloprid (common neonicotinoid; IMI) exposure would make *Drosophila melanogaster* (an insect model for the honey bee) more susceptible to bacterial pathogens, heat stress, and intestinal dysbiosis. Our results suggested that the immune deficiency (Imd) pathway is necessary for *D*. *melanogaster* survival in response to IMI toxicity. IMI exposure induced alterations in the host-microbiota as noted by increased indigenous *Acetobacter* and *Lactobacillus* spp. Furthermore, sub-lethal exposure to IMI resulted in decreased *D*. *melanogaster* survival when simultaneously exposed to bacterial infection and heat stress (37 °C). This coincided with exacerbated increases in *TotA* and *Dpt* (Imd downstream pro-survival and antimicrobial genes, respectively) expression compared to controls. Supplementation of IMI-exposed *D*. *melanogaster* with *Lactobacillus plantarum* ATCC 14917 mitigated survival deficits following *Serratia marcescens* (bacterial pathogen) septic infection. These findings support the insidious toxicity of neonicotinoid pesticides and potential for probiotic lactobacilli to reduce IMI-induced susceptibility to infection.

## Introduction

To remain economically feasible, agricultural production commonly relies on pesticide use to maintain high crop yield. Widespread implementation of transgenic plants has attempted to reduce the use of pesticides, however supplementary delivery methods are often required for complete pest control^1, 2^. Consequently, foliar spray-based application of imidacloprid (a common neonicotinoid; IMI) and other insecticides continues to be necessary and thus increases the risk of honey bee (*Apis mellifera*) exposure to pesticides and hive contamination^3^. Declines in honey bee health are problematic due to their role as critical pollinators for roughly 35% of the global food crop^4^, and thus, are largely responsible for the world’s food supply and economy.

Colony collapse disorder has been attributed to increased exposure of honey bees to a combination of pesticides^5^ and pathogens^6^ and increased habitat loss^7^. Neonicotinoids are currently the most widely used insecticides in the world, owing largely to affordability, flexible application, and long-lasting systemic activity in plant tissue^8^. In addition, neonicotinoids have a relatively low toxicity profile toward vertebrates, unlike organophosphate and carbamate insecticides^9^. However, neonicotinoid insecticides appear to be particularly toxic to bees^5, 10^. These compounds are insect selective nicotinic acetylcholine receptor agonists, which have been associated with bee growth abnormalities^10^, motor deficiencies^5^, neurologic abnormalities^11^, and/or death^5^. The commonly used neonicotinoids, IMI and clothianidin, have also been shown to suppress immune function and consequently increase susceptibility of honey bees to viral and fungal infections^12^. Thus, neonicotinoids may be more problematic than anticipated due to their immunosuppressive effects at concentrations below the threshold for neurotoxicity. The current dilemma facing the agriculture industry is how to resolve the issue of preventing bee decline, while mitigating crop losses associated with pest infestations.

*Drosophila melanogaster* (fruit fly) is a convenient insect organism to model pesticide-induced toxicity in bees^12, 13^. Both species are profoundly affected by IMI in terms of toxicity and immunosuppressive effects^12^. Fruit flies and honey bees have a core microbiota composed of *Lactobacillus* and *Acetobacter* spp., with *Lactobacillus plantarum* a predominant member in both hosts^14–16^. Lactobacilli have been shown to induce positive effects on *D*. *melanogaster* and honey bee immune function^17, 18^ and growth/development^19^. In particular, prophylactic lactobacilli supplementation has shown to mitigate lethal *Serratia marcescens* and *Pseudomonas aeruginosa* infections in *D*. *melanogaster* through an uncharacterized mechanism^20^. The use of *D*. *melanogaster* genetic tools provides the opportunity to evaluate the effect of microbiota manipulation on insect responses to pesticides and environmental stressors prior to verification in honey bees^21–23^.

Innate immunity of insects is largely orchestrated by the Toll, immune deficiency (Imd), and Janus kinase and two Signal Transducer and Activator of Transcription (JAK/STAT) pathways^24–27^. The JAK/STAT and Imd pathway enable insects to adapt and survive environmental challenges such as heat stress, infection, paraquat (herbicide), and UV exposure^15, 16^. These pathways are both regulated by the downstream transcription factor, Relish^19^. Relish a key signaling hub for coordinating host physiological responses to bacteria through the Imd/nuclear factor kappa-light-chain-enhancer of activated B cells (Imd/NF-kB) pathway^17^. Relish is best characterized as a downstream transcription factor that elicits antimicrobial peptide immune responses to diaminopimelic acid (DAP)-type peptidoglycan activation of peptidoglycan recognition protein (PGRP)-LE in the midgut and PGRP-LC in the hindgut and foregut^28^. *Drosophila melanogaster* with Imd pathway loss of function phenotypes are significantly more susceptible to Gram-negative bacterial infections^29–31^. The indigenous *D*. *melanogaster* microbiota—*Lactobacillus* spp. in particular—are known to activate *Relish* in the gut without downstream antimicrobial peptides due to negative regulation^17, 32, 33^. However, the implications of this interaction between intestinal Relish and the fruit fly microbiota is still poorly understood^32^. It is also largely unknown how exogenous microorganisms, such as probiotic bacteria, modulate the Imd pathway.

Therefore, since the Imd-Relish pathway is critical for mediating *D*. *melanogaster* survival responses to noxious stimuli^34^, it was hypothesized that probiotic lactobacilli could mitigate IMI-induced immune deficits in *D*. *melanogaster*. The main objectives of this study were to 1) determine if IMI exposure affected the ability of *D*. *melanogaster* to fight bacterial pathogens, handle heat stress, and regulate microbial populations, and 2) determine if probiotic lactobacilli could mitigate IMI-induced susceptibility to bacterial infection in *D*. *melanogaster*.

## Results

### IMI exposure in *D*. *melanogaster* results in dose-dependent toxicity

**Figure 1:**
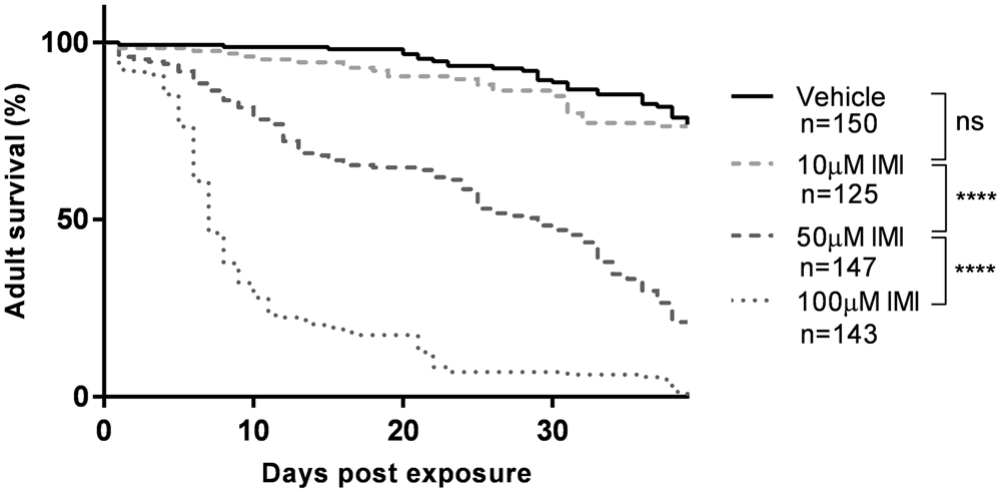
IMI exposure in *D*. *melanogaster* results in dose dependent toxicity. Survival curves for newly eclosed wildtype Canton-S flies fed food containing vehicle or food containing varying concentrations of IMI. Data are displayed from at least 5 independent experiments (20–25 flies each group per experiment). All statistical symbols are representative of comparison made using the log-rank (Mantel-Cox) test. ****p < 0.0001. ns = not significant.

To determine an appropriate sub-lethal dosage of IMI for further experimentation, wildtype (WT) Canton-S flies were exposed to varying concentrations of IMI. WT Canton-S flies reared on food containing 10 µM IMI showed no significant differences in overall survival (log rank [Mantel-Cox], chi-square = 0.5069, degrees of freedom [df] = 1, *P = *0.4765; Fig. 1) compared to vehicle-exposed controls. Similar trends were seen in *Rel* ^−/−^ *D*. *melanogaster* (Supp. Fig. 3). This indicated that exposure to 10 µM IMI represented a sub-lethal chronic dosage in WT Canton-S flies.

### IMI exposure results in an increased abundance of indigenous *Acetobacter* and *Lactobacillus* spp. in *D*. *melanogaster*

**Figure 2:**
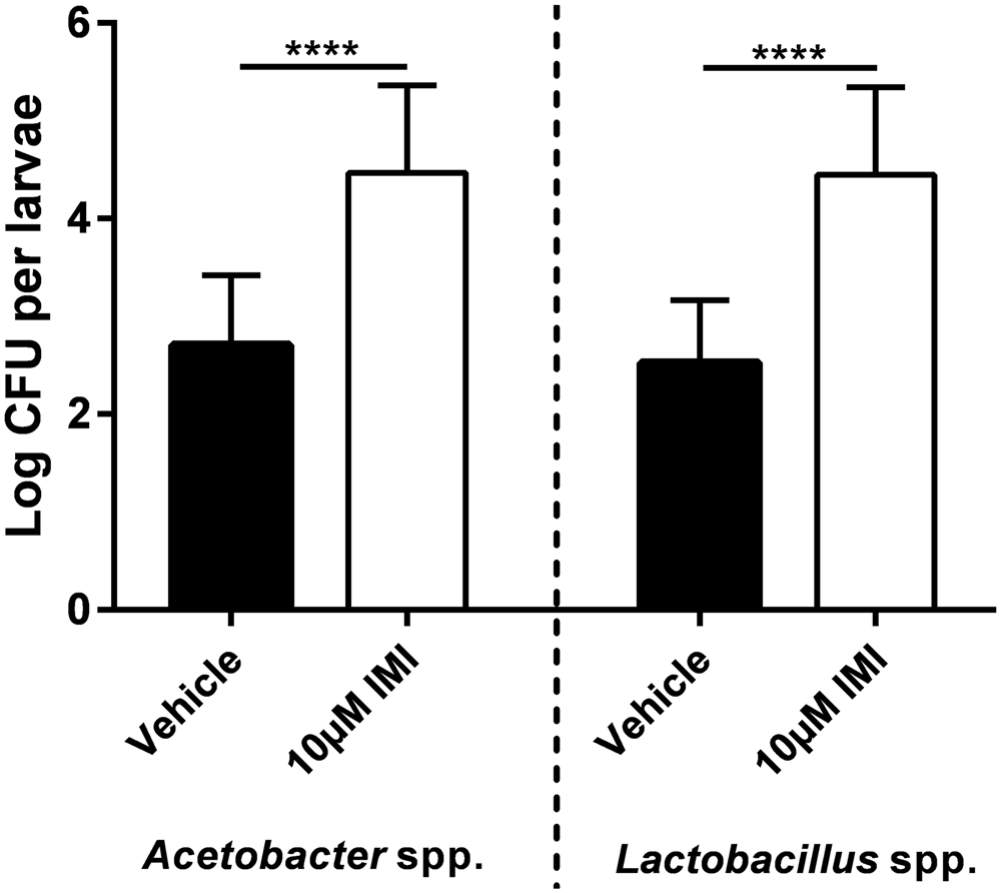
IMI exposure results in an increased abundance of indigenous *Acetobacter* and *Lactobacillus* spp. in *D*. *melanogaster*. Third-instar larvae that were reared on food containing vehicle or 10 μM IMI were surface-sterilized, homogenized, plated on selective medium, and incubated at 37 °C. Colony forming units (CFU) were subsequently enumerated after 48 h of incubation. Mean ± standard deviation (unpaired, two-tailed t-tests) of 3 independent experiments (n = 15 for each group). ****p < 0.0001.

Xenobiotics, including environmental toxins such as pesticides, often come into direct contact with the intestinal microbiota via oral exposure, and can alter its composition and/or function^35, 36^. Thus, we sought to determine if IMI exposure could alter the composition of dominant bacterial genera in *D*. *melanogaster*. Third-instar WT Canton-S larvae reared on 10 µM IMI-containing media harboured significantly more *Acetobacter* and *Lactobacillus* spp. (t tests, t = 5.933, df = 28, *P* < 0.0001 and t = 6.734, df = 28, *P* < 0.0001, respectively) compared to vehicle controls (Fig. 2). However, there was no significant difference in the ratio of *Acetobacter* to *Lactobacillus* spp. between IMI-exposed and vehicle flies. This suggested that IMI could be affecting the innate immune function of *D*. *melanogaster* and thus their ability to regulate microbiota populations.

It was found that antibiotic-treated WT flies exposed to lethal concentrations of imidacloprid (100 µM) had significantly increased overall survival (log-rank [Mantel-Cox], chi-square = 25.54, *P* < 0.0001) and less early time point deaths (Gehan-Breslow-Wilcoxon test, chi-square = 27.09, *P* < 0.0001) in comparison to WT vehicle flies exposed to imidacloprid but not treated with antibiotics (Supp. Fig. 1). *Rel* ^−/−^ mutants exposed to 100 µM imidacloprid followed a similar trend with antibiotic treated flies having significantly increased overall survival (log-rank [Mantel-Cox], chi-square = 11.75, *P* = 0.0006) and reduced early time point deaths (Gehan-Breslow-Wilcoxon test, chi-square = 10.21, *P* = 0.0014) compared to flies not treated with antibiotics. These results suggested that artificially regulating microbial populations could mitigate the effects of IMI.

### The Imd pathway is necessary to mitigate IMI-induced toxicity in *D*. *melanogaster*

**Figure 3:**
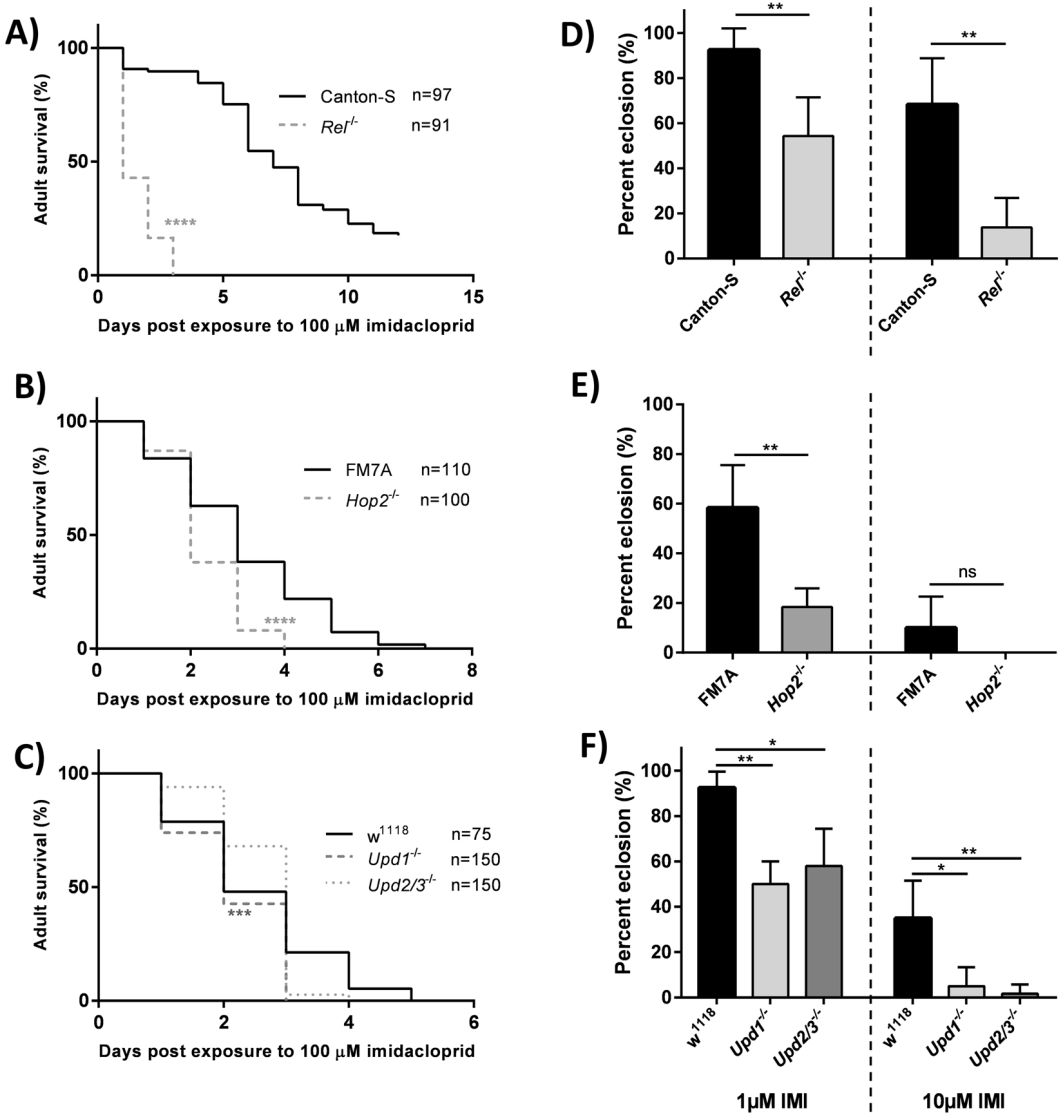
The Imd pathway is necessary to mitigate IMI-induced toxicity in *D*. *melanogaster*. (**A**–**C**) Survival curves for newly eclosed *D*. *melanogaster* Imd pathway mutant flies (*Rel* ^−/−^, *Hop2* ^−/−^, *Upd1* ^−/−^, *Upd2/3* ^−/−^) exposed to 100 μM IMI compared to their wildtype background controls (Canton-S, FM7A, and w^1118^, respectively). Data are displayed from at least 3 independent experiments (20–25 flies for each group per experiment). Statistical analyses shown are from log-rank (Mantel-Cox) tests. (**D**–**F**) First-instar Imd pathway mutant larvae and their respect background controls were exposed to 1 μM and 10 μM IMI and percentage of larvae that subsequently eclosed. Data are means ± standard deviations (Mann-Whitney tests [D,E]; Kruskal-Wallis tests [F]) of results from 5 biological replicates (10 larvae per biological replicate; n = 5 for each group). *p < 0.05 **p < 0.01, ***p < 0.001, ****p < 0.0001. ns = not significant.

To test if the Imd pathway was necessary for resisting IMI-induced toxicity, loss-of-function mutants for various genes involved in Imd signalling pathway were assessed for survival and developmental deficits following IMI exposure. *Rel* ^−/−^ flies exhibited significantly reduced overall survival (log rank [Mantel-Cox], chi-square = 163.6, df = 1, *P* < 0.0001) and more early time point deaths (Gehan-Breslow-Wilcoxon test, chi-square = 127.9, df = 1, *P* < 0.0001) when exposed to 100 µM IMI compared to WT Canton-S flies (Fig. 3A). *Hop2* ^−/−^ flies also exhibited significantly reduced overall survival (log rank [Mantel-Cox], chi-square = 26.39, df = 1, *P* < 0.0001) and greater early time point deaths (Gehan-Breslow-Wilcoxon test, chi-square = 15.95, df = 1, *P* < 0.0001) when exposed to 100 µM IMI compared to background FM7A flies (Fig. 3B). *Upd1* ^−/−^ flies had significantly reduced overall survival (log rank [Mantel-Cox], chi-square = 10.91, df = 1, *P* = 0.0010) and greater early time point deaths (Gehan-Breslow-Wilcoxon test, chi-square = 4.789, df = 1, *P* = 0.0286) when exposed to 100 µM IMI compared to background w^1118^ flies. *Upd2/3* ^−/−^ flies showed a trend of greater early time point deaths (Gehan-Breslow-Wilcoxon test, chi-square = 2.012, df = 1, *P = *0.1561), but not in overall survival (log-rank [Mantel-Cox], chi-square = 0.0355, *P* = 0.8506) compared to background w^1118^ flies (Fig. 3C).

To determine if Imd pathway loss-of-function larvae were more susceptible to IMI-induced developmental deficits, first-instar larvae were incubated on media containing 1 µM IMI, 10 µM IMI, or vehicle and monitored for subsequent eclosion (emergence of adult flies from pupae). *Rel* ^−/−^ mutant larvae demonstrated significantly impaired eclosion when exposed to both 1 µM and 10 µM IMI (Mann-Whitney tests, U = 0.5, *P* = 0.0091 and U = 1, *P* = 0.0013, respectively) compared to Canton-S larvae (Fig. 3D). *Hop2* ^−/−^ mutant larvae also had significantly impaired eclosion at 1 µM and a trend towards impairment at 10 µM IMI exposure concentrations (Mann-Whitney tests, U = 0.5, *P* = 0.0043 and U = 12, *P* = 0.0549, respectively) compared to background FM7A larvae (Fig. 3E). Furthermore, *Upd1* ^−/−^ and *Upd2/3* ^−/−^ mutant larvae demonstrated significantly impaired eclosion when exposed to 1 µM IMI (Kruskal-Wallis, *P* = 0.0023 and *P* = 0.0255, respectively) and 10 µM IMI (Kruskal-Wallis, *P* = 0.0229 and *P* = 0.0047, respectively) compared to background w^1118^ larvae (Fig. 3F). Together, these results suggested that the Imd pathway is necessary for mitigating IMI-induced toxicity.

### IMI exposed *D*. *melanogaster* respond similarly to heat stress as Imd pathway mutants

**Figure 4:**
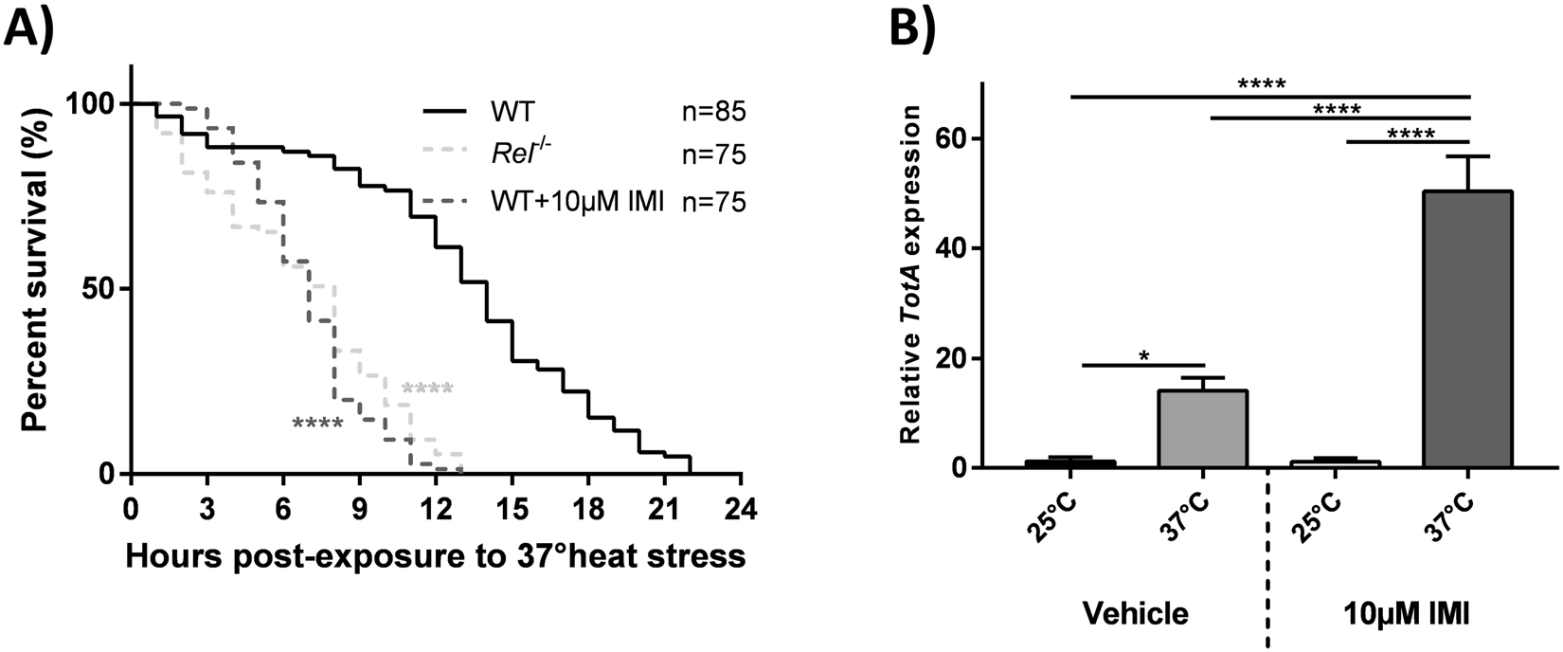
IMI exposed *D*. *melanogaster* respond similarly to heat stress as Imd pathway mutants. (**A**) Survival curves for newly eclosed Imd pathway mutants (*Rel* ^−/−^) and WT Canton-S exposed to heat stress (37°) with or without concurrent exposure to IMI. Data are displayed from at least 3 independent experiments (15–25 flies reared in separate food vials on different occasions for each experiment). Statistical analyses are representation of comparisons made to heat-stressed WT Canton-S controls using the log-rank (Mantel-Cox) test. (**B**) *TotA* gene expression of newly eclosed *D*. *melanogaster* that were heat stressed (37°) with or without concurrent exposure to IMI compared to non-heat stressed (25°) controls. All samples were taken 6 h after experimental start time. Gene expression was quantified using RT-qPCR and is relative to vehicle flies not exposed to heat stress. Means ± standard deviations (two-way ANOVA) from 3 biological replicates (each consisting of 10 flies) with triplicate technical repeats are shown. ****p < 0.0001.

Relish has been reported to have pleiotropic effects outside immune regulation, including the promotion of survival in response to environmental heat stress^34^. *Rel* ^−/−^ flies were used to comparatively illustrate similarities in the heat stress response between IMI-exposed WT Canton-S flies and non-exposed Imd-pathway mutants. Following heat stress, *Rel* ^−/−^ mutant flies had significantly shorter overall survival (log rank [Mantel-Cox], chi-square = 79.51, df = 1, *P* < 0.0001) and more early time point deaths (Gehan-Breslow-Wilcoxon test, chi-square = 60.25, df = 1, *P* < 0.0001) compared to WT Canton-S flies (Fig. 4A). Notably, IMI-exposed WT Canton-S flies responded similarly to heat stress as non-exposed *Rel* ^−/−^ flies, exhibiting significantly reduced overall survival (log rank [Mantel-Cox], chi-square = 89.8, df = 1, *P* < 0.0001) and more early time point deaths (Gehan-Breslow-Wilcoxon test, chi-square = 64.74, df = 1, *P* < 0.0001) compared to WT Canton-S vehicle flies that were exposed to heat stress but not IMI (Fig. 4A).

To determine the effects of IMI on Imd pathway regulation, gene expression of *TotA* (Imd downstream pro-survival gene) was measured. Heat stress significantly increased *TotA* expression in all WT Canton-S flies exposed to 37 °C heat stress when compared to 25 °C controls, whether exposed to IMI or not (Fig. 4B). However, there was a significant upregulation of *TotA* expression in IMI-exposed WT Canton-S flies subjected to 37 °C heat stress compared to vehicle WT Canton-S flies at 37 °C (two-way ANOVA, *P* < 0.0001; Fig. 4B). These results suggested exposure to IMI reduced overall survival and exacerbated Imd pathway activation during heat stress.

### IMI-exposed *D*. *melanogaster* are more susceptible to septic infection with *S*. *marcescens*

**Figure 5:**
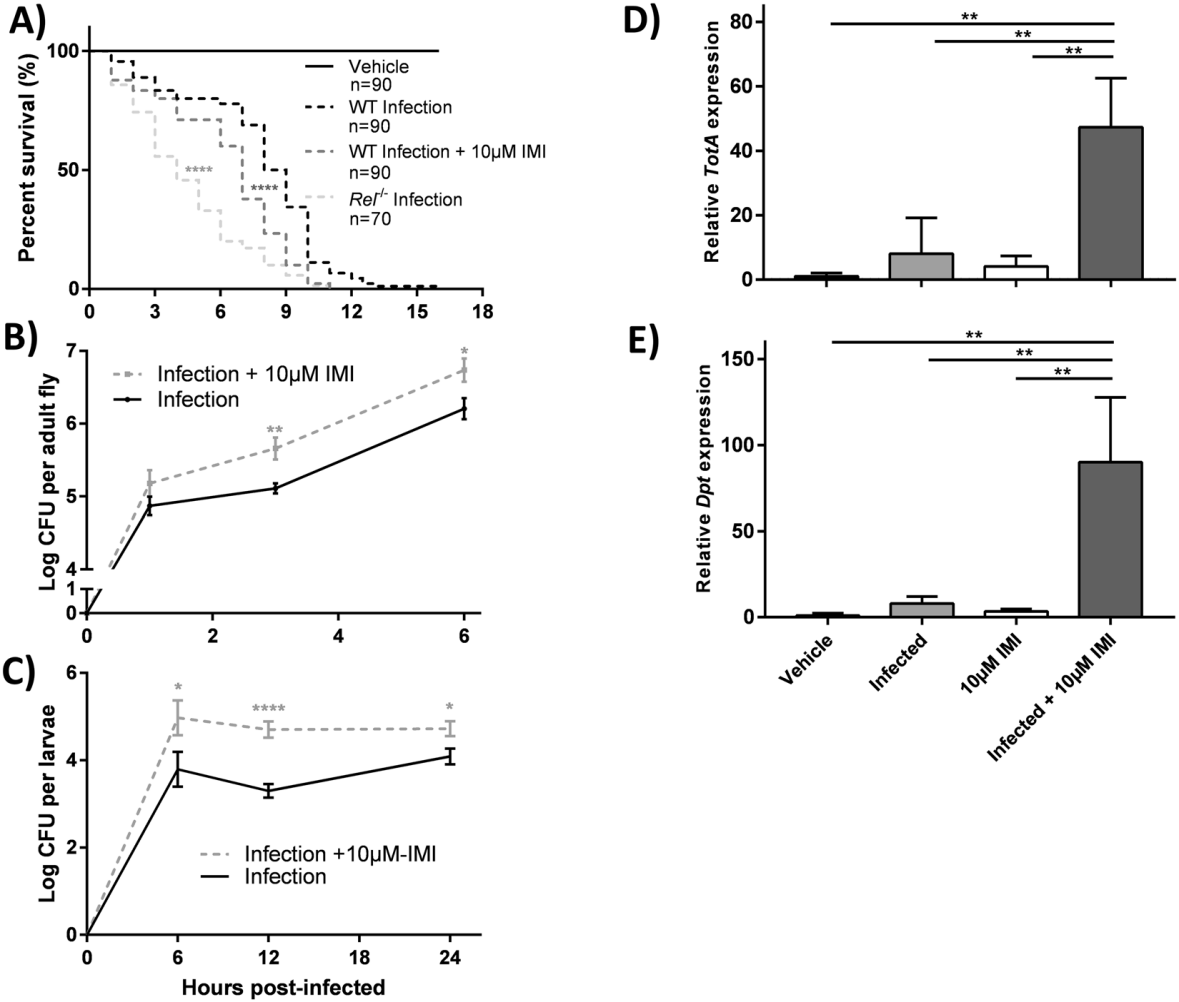
IMI-exposed *D*. *melanogaster* are more susceptible to septic infection with *Serratia marcescens*. (**A**) Survival curves for newly eclosed Imd pathway mutant (*Rel* ^−/−^) and WT Canton-S flies subjected to septic infection (*S*. *marcescens* NCIMB 11782) with or without concurrent exposure to IMI. All statistical symbols are representative of comparison made to infected WT Canton-S controls using the log-rank (Mantel-Cox) test. (**B**) Pathogen load of *S*. *marcescens* NCIMB 11782 during septic infection of WT flies was determined by plating surface-sterilized whole fly homogenates on LB agar medium. Colony forming units (CFU) per fly obtained at each time point represents the mean ± standard deviation (unpaired, two-tailed t-tests) of 9 biological replicates (n = 36 for each group). (**C**) Pathogen load of *S*. *marcescens* Db11 during oral infection of WT larvae was determined by plating surface-sterilized whole larvae homogenates on LB with 100 μg/ml streptomycin medium. CFU per larvae obtained at each time point represents the mean ± standard deviation (unpaired, two-tailed t-tests) of 9 biological replicates for each time point (n = 36 total for each group). (**D**,**E**) *TotA and Dpt* gene expression of newly eclosed WT flies that were subjected to septic injury with or without *S*. *marcescens* NCIMB 11782 infection and with or without concurrent exposure to IMI. All samples were taken 6 h after experimental start time. Gene expression was quantified by RT-qPCR and is relative to vehicle flies that were subjected to septic injury with a sterile needle. Means ± standard deviations (two-way ANOVA) from 3 biological replicates (each consisting of 10 flies) with triplicate technical repeats are shown. *p < 0.05 **p < 0.01, ***p < 0.001, ****p < 0.0001.

Since the Imd pathway is critical to responding to Gram-negative bacterial infections^17^, similar comparative experiments were performed to elucidate the effect of IMI exposure on pathogenicity of *S*. *marcescens* NCIMB 11782 to *D*. *melanogaster* (common infection model). WT Canton-S and *Rel* ^−/−^ flies that were subjected to septic infection displayed median survivals of 8.5 and 4 h, respectively. *Rel* ^−/−^ flies that were infected had significantly decreased overall survival (log rank [Mantel-Cox], chi-square = 46.26, df = 1, *P* < 0.0001) and more early time point deaths (Gehan-Breslow-Wilcoxon test, chi-square = 42.63, df = 1, *P* < 0.0001) compared to infected WT Canton-S flies (Fig. 5A). Alternatively, IMI-exposed WT Canton-S subjected to septic infection exhibited an intermediate phenotype with a median survival of 7 h. Notably, infected IMI-exposed WT Canton-S flies had significantly reduced overall survival (log rank [Mantel-Cox], chi-square = 20.84, df = 1, *P* < 0.0001) and more early time point deaths (Gehan-Breslow-Wilcoxon test, chi-square = 16.87, df = 1, *P* < 0.0001) compared to infected vehicle-exposed WT Canton-S flies (Fig. 5A).

IMI-exposed adult WT Canton-S flies were found to have significantly increased pathogen loads at 3 and 6 h (t tests, t = 3.306, df = 16, *P* = 0.0045 and t = 2.472, df = 16, *P* = 0.0250, respectively) after *S*. *marcescens* NCIMB11782 septic infection compared to infected vehicle-exposed WT Canton-S flies (Fig. 5B). To evaluate if oral infection resulted in similar trends, third-instar larvae were orally infected with *S*. *marcescens* Db11. IMI exposure resulted in significantly increased pathogen burden at 6, 12, and 24 h (t tests, t = 2.083, df = 35, *P = *0.0446 and t = 5.629, df = 24, *P* < 0.0001, and t = 2.504, df = 23, *P* = 0.0198 respectively; Fig. 5C) in WT Canton-S larvae relative to vehicle-exposed controls.

IMI-exposed WT Canton-S flies that were subjected to septic infection with *S*. *marcescens* NCIMB 11782 exhibited significantly elevated *TotA* gene expression compared to WT Canton-S flies that were infected but not exposed to IMI (two-way ANOVA, *P* = 0.0046; Fig. 5D). Likewise, *Dpt* (Imd downstream antimicrobial peptide) expression was also significantly increased in infected WT Canton-S flies exposed to IMI compared to infected WT Canton-S flies not exposed to IMI (two-way ANOVA, *P* = 0.0045; Fig. 5E). No significant difference was seen in *Dpt* expression between 10 µM IMI-exposed and vehicle-fed *D*. *melanogaster* (Supp. Fig. 2). These results suggested IMI exposure increased susceptibility to a bacterial pathogen and exacerbated IMI-induced Imd pathway activation in response to infection.

### Lp39 can tolerate, but not bind or metabolize IMI

**Figure 6:**
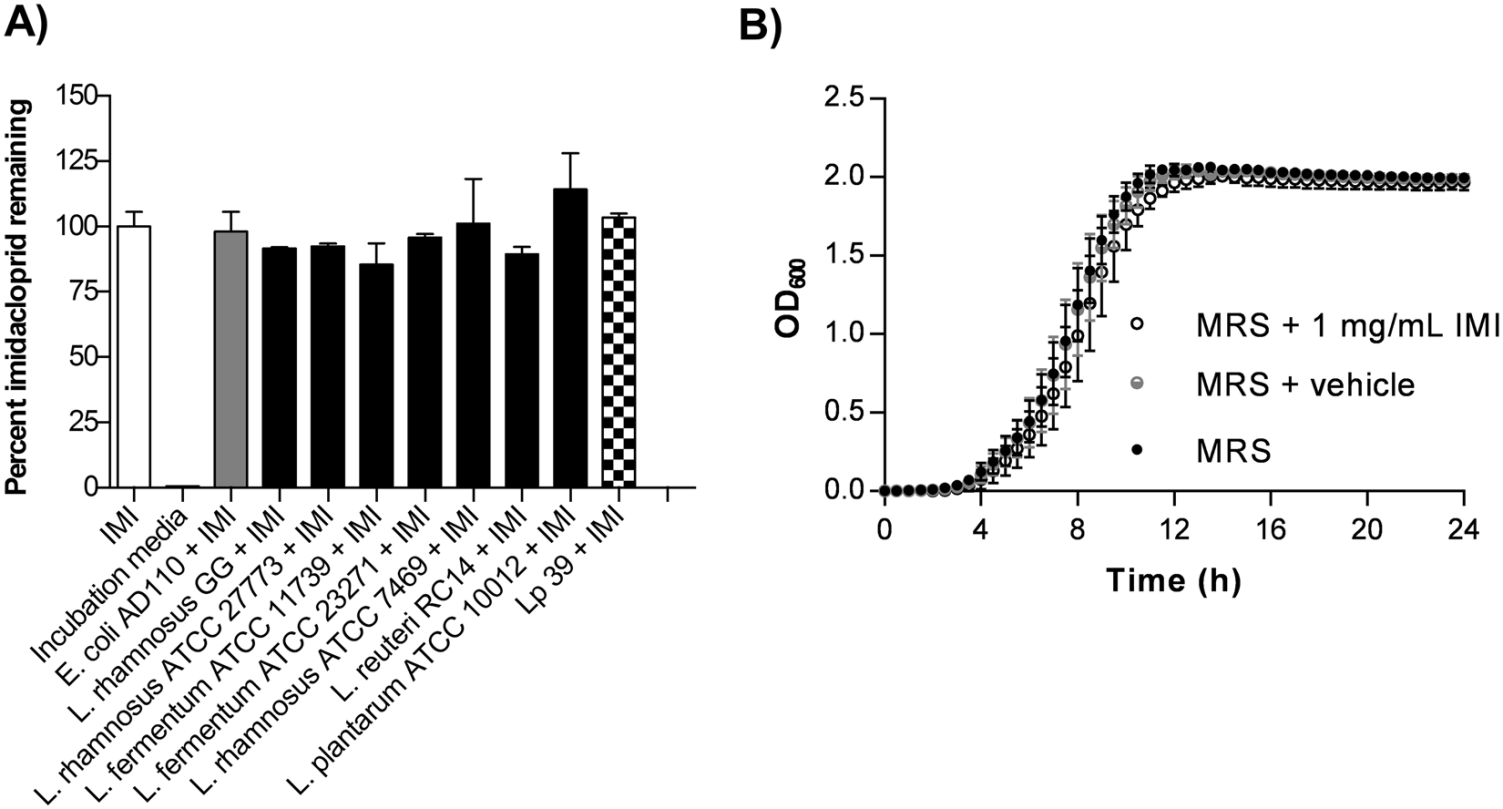
Lp39 can tolerate, but not bind or metabolize IMI. (**A**) Percent imidacloprid (IMI) was determined in stationary phase live bacterial cultures relative to pesticide-only controls following 24 h co-incubations in 50 mM HEPES. Data are depicted as means ± standard deviations (one-way ANOVA) of 2 independent experiments (2 biological replicates each). (**B**) Growth curves of Lp39 in MRS and MRS supplemented with vehicle or imidacloprid (IMI). Data are depicted as means ± standard deviations of 3 biological replicates with triplicate technical replicates.

Several species of *Lactobacillus* were screened to identify a candidate that did not directly interact with IMI. All *Lactobacillus* species that were tested failed to demonstrate any notable metabolism or binding of IMI (0.1 mg/ml ≈ 391.1 µM) during 24 h of co-incubation (Fig. 6A). It was determined Lp39 could grow unimpaired at high concentrations of IMI (1 mg/mL ≈ 3911 µM) with no significant differences in growth compared to vehicle (Fig. 6B). Thus, Lp39 was chosen for further investigation due to its probiotic and immunostimulatory properties in both *D*. *melanogaster* and honey bees^37, 38^.

### Lp39 supplementation mitigates IMI-induced *D*. *melanogaster* survival deficits following infection

**Figure 7:**
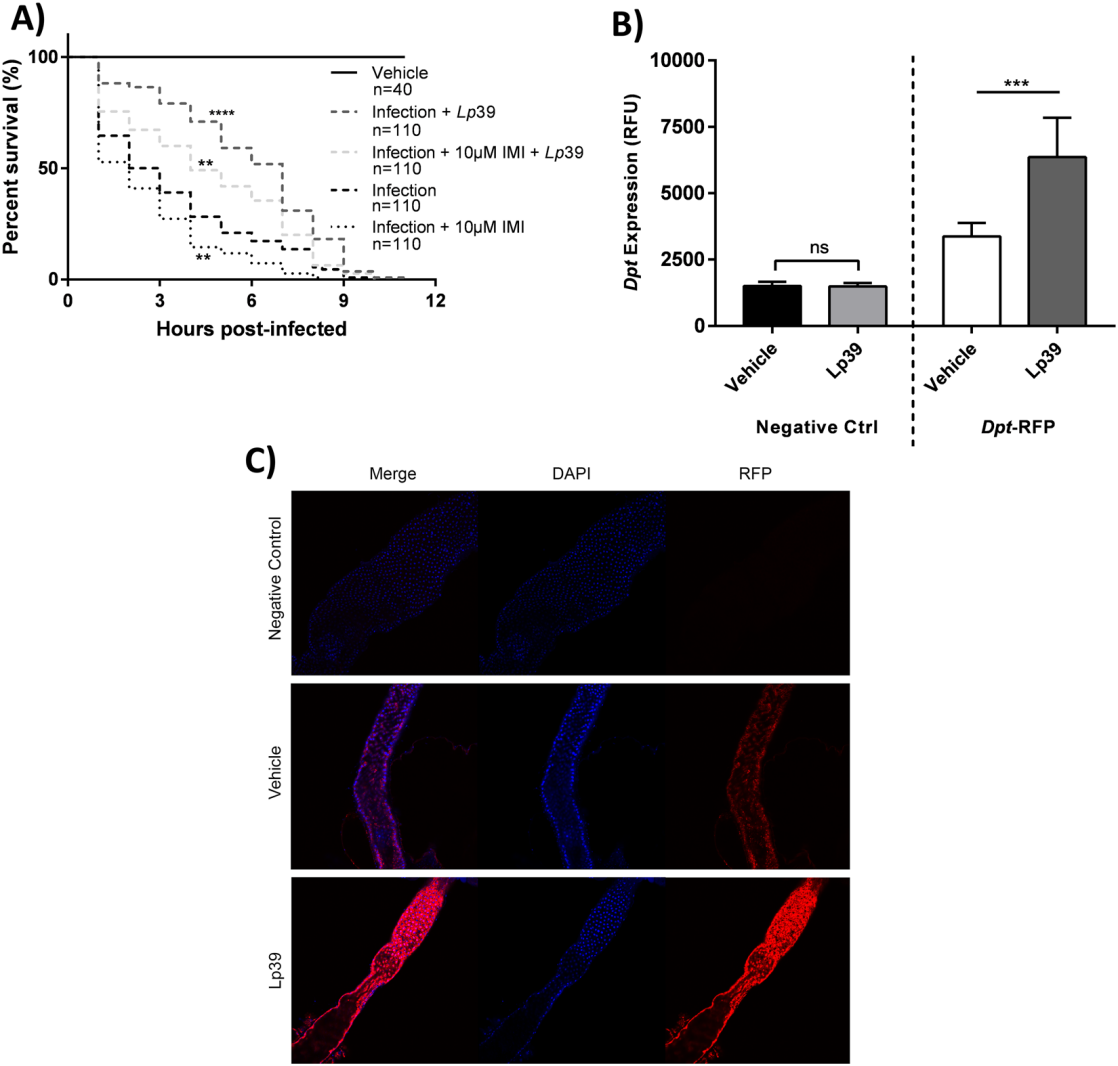
Lp39 supplementation mitigates IMI-induced *D*. *melanogaster* survival deficits following infection. (**A**) Survival curves for newly eclosed WT Canton-S flies that were subjected to septic infection with *Serratia marcescens* NCIMB 11782 while concurrently exposed to IMI and/or supplemented with Lp39 separately or in combination, compared to infected WT-Canton control and vehicle (septic injury alone). All statistical symbols are representative of comparison made to infected WT Canton-S controls. Statistical significance was determined using the log-rank (Mantel-Cox) test. (**B**) *Dpt* expression of newly eclosed *Dpt*-RFP reporter flies was determined following 15 h oral supplementation of Lp39 or vehicle. WT Canton-S flies were used as a negative control. Gene expression was quantified by fluorescence intensity using a microplate reader. Mean ± standard deviations (two-way ANOVA) from 7 biological replicates (10 flies each replicate) per group are shown. (**C**) Newly enclosed *Dpt*-RFP and negative control WT Canton-S flies were orally supplemented with Lp39 or vehicle for 15 h. Midguts were dissected, nuclear counterstained with Hoechst 33342, trihydrochloride, tetrahydrate and viewed by confocal microscopy. Scale bar = 20 µm. **p < 0.01, ***p < 0.001, ****p < 0.0001.

WT Canton-S flies that were subjected to septic infection with *S*. *marcescens* NCIMB 11782 had a median survival of 2.5 h, which was increased to 7 h when supplemented with Lp39. Notably, flies in these experiments died more rapidly to infection in comparison to earlier septic infection experiments. These discrepancies are believed to be due to uncontrollable biological variations that occur in different batches of flies. Importantly though, trends between IMI-exposed infected flies and infected vehicle-fed controls remained similar to previous findings. Lp39 supplementation significantly increased overall survival (log rank [Mantel-Cox], chi-square = 33.09, df = 1, *P* < 0.0001) and reduced early time point deaths (Gehan-Breslow-Wilcoxon test, chi-square = 40.71, df = 1, *P* < 0.0001) in infected flies (Fig. 7A). Similarly, IMI-exposed WT Canton-S flies had a median survival of 2 h after septic infection, which increased to 4 h when supplemented with Lp39. Lp39 significantly increased overall survival (log rank [Mantel-Cox], chi-square = 35.77, df = 1, *P* < 0.0001) and reduced early time point deaths (Gehan-Breslow-Wilcoxon test, chi-square = 28.66, df = 1, *P* < 0.0001) in IMI-exposed and infected flies. These findings suggested that Lp39 reduced *D*. *melanogaster* susceptibility to infection regardless of concurrent IMI exposure, although to a lesser extent (Fig. 7A).

To determine the effect of Lp39 supplementation on Imd pathway activity, *Dpt*-RFP flies were used to determine *Dpt* gene expression. *Dpt*-RFP flies orally-supplemented with Lp39 had significantly increased expression of *Dpt* compared to vehicle-fed *Dpt*-RFP flies (two-way ANOVA, *P* < 0.0001; Fig. 7B). No significant difference was seen in Lp39-supplemented negative control WT Canton-S flies compared to vehicle (two-way ANOVA, *P = *0.9992). Furthermore, qualitative observation of dissected midguts demonstrated increased *Dpt*-RFP expression in flies that had been orally-supplemented with Lp39 relative to vehicle-fed controls (Fig. 7C).

## Discussion

This study demonstrated that the Imd pathway was necessary for *D*. *melanogaster* larvae and adult pro-survival in response to IMI. Relish loss-of-function (*Rel* ^−/−^) flies and larvae had significantly increased susceptibility to IMI-induced toxicity, compared to WT Canton-S controls. In addition, flies and larvae with loss-of-function genotypes in factors upstream of Relish, including *Hop2* ^−/−^, *Upd1* ^−/−^, and *Upd2/3* ^−/−^, displayed significantly increased susceptibility to IMI compared to their respective genetic backgrounds. The honey bee has several homologues with high amino acid sequence similarity to the *D*. *melanogaster* Relish protein such as the predicted nuclear factor NFkB p110 subunit isoform X1 (XP_006562282.1), dorsal (NP_001011577.1), and Relish itself (ACT66913.1). These findings and the highly-conserved nature of innate immunity suggest that translation of findings using this *D*. *melanogaster* model are relevant to honey bees and will be amenable for future investigation of pesticide interventions in honey bees^18, 39^.

Neonicotinoid pesticides, such as IMI, have been shown to downregulate NF-kB insect immune responses at sub-lethal concentrations due to upregulation of the NF-kB negative regulator gene *CG1399* present in *D*. *melanogaster* ^12^. Compared to non-exposed controls, WT Canton-S flies reared on IMI experienced significantly increased mortality when exposed to 37 °C heat stress or *S*. *marcescens* septic infection (Figs 4A and 5A, respectively). WT Canton-S larvae and flies reared on IMI also demonstrated significantly increased *S*. *marcescens* pathogen burden following oral and septic infection relative to controls, respectively. This increased pathogen burden in IMI exposed WT Canton-S flies was associated with significantly increased *Dpt* expression (antimicrobial peptide), but it was not protective against early mortality from septic infection. This finding is explained, in part, by the fact that *S*. *marcescens* has been shown to resist innate immune response in *D*. *melanogaster* ^30^. Furthermore, flies reared on IMI demonstrated significant upregulation of the JAK/STAT stress response gene *TotA* following heat stress and *S*. *marcescens* septic infection (Figs 4B and 5D). *TotA* induction is critically important for responding to a variety of stressful stimuli such as bacterial infection and heat stress^21, 22^. Imd-activation and overall stress was synergistically higher when IMI and other harmful stimuli were encountered simultaneously by *D*. *melanogaster*. These results are supported in other insect studies, which have shown reduction in melanotic encapsulation of IMI-exposed Asian longhorned beetles (*Anoplophora glabripennis*) infected with the entomopathogenic fungus, *Metarhizium brunneum*. In addition, increased microsporidia *Nosema apis* pathogenicity^40, 41^ and deformed wing virus replication^12^ have been reported in neonicotinoid-exposed honey bees.

Although IMI has been shown to alter microbial ecosystems in soil^42, 43^, the effect of neonicotinoids on the insect intestinal microbiota has yet to be elucidated. This study demonstrated that WT Canton-S flies exposed to IMI had significantly increased absolute abundance of intestinal *Acetobacter* and *Lactobacillus* genera. These findings are suggestive of a potentially altered immune function, as commensal-gut mutualism is dependent on antimicrobial peptide regulation by the innate immune system^32, 44^. Since the gut ecosystem is important for regulating numerous aspects of insect physiology including growth^19^, lifespan^45^, and mating behavior of insects^46^, the consequences of IMI-induced disruption of the insect microbiota merits further study.

Imd pathway activation can prime *D*. *melanogaster* to mitigate infection^20, 47^, thus we hypothesized that targeting this pathway with beneficial microorganisms could mitigate dysregulated immunity observed in *D*. *melanogaster* during IMI challenge. Administration of probiotic *L*. *plantarum* has been shown to increase survival, lessen gut epithelial damage, and reduce bacterial translocation associated with *S*. *marcescens* infection^48^. The present finding that oral Lp39 supplementation significantly mitigated IMI-induced susceptibility of WT Canton-S flies to *S*. *marcescens* septic infection mortality relative to unsupplemented controls, suggest that Lp39-mediated protection was likely host-mediated, as there would have been minimal direct interaction between *S*. *marcescens* (septic infection) and Lp39 (oral supplementation). Interestingly, Lp39 supplemented WT Canton-S flies exposed to IMI also displayed significantly better overall survival and less early time point deaths compared to flies not exposed to IMI following *S*. *marcescens* infection. This could be partially explained by the finding of significantly increased expression of the Imd pathway response antimicrobial gene, *Dpt*, following Lp39 supplementation. This aligns with past studies demonstrating the immunogenic potential of lactobacilli DAP-type peptidoglycan in modulating the Imd pathway^49–51^.

Since the honey bee genome has about half as many glutathione-S-transferases, cytochrome P450 (Cyp) monooxygenases, and carboxyl/cholinesterases as *D*. *melanogaster* ^52^, it is challenging to directly extrapolate our findings. This is important since these enzymes are involved in detoxification of IMI. In particular, the global spread of Cyp6g1 overexpression in *D*. *melanogaster* has resulted in a high level of resistance to a variety of insecticides including IMI^53^. This study determined that 10 µM IMI (approximately 50x higher than what is generally observed in agricultural settings)^8^ represented a sub-lethal dosage in *D*. *melanogaster*. However, the observed deficits of IMI-exposed *D*. *melanogaster* to additional stressors (e.g. infection, heat) are likely relevant to honey bees at environmental concentrations due to their previously reported sensitivity to neonicotinoid pesticides^3, 5, 10^.

In summary, this study has shown that: 1) the Imd pathway was necessary for promoting insect survival in response to IMI toxicity, 2) IMI exposure altered the insect microbiota, and 3) IMI exposure exacerbated insect susceptibility to both heat stress and bacterial infection. Furthermore, Lp39 mitigated IMI-induced susceptibility to a bacterial pathogen in *D*. *melanogaster*. These findings provide evidence for the insidious insect toxicity of neonicotinoid pesticides in combination with other environmental stressors and illustrate the prophylactic potential of lactobacilli to combat some of the predicted causes of colony collapse disorder. The extension of these findings to honey bees is promising given that colony supplementation with lactobacilli is affordable, feasible, and has already been shown to benefit honey bee colony growth^54^, microbiota composition^55^, and antimicrobial defense^18, 20^.

## Methods

### Chemicals

Imidacloprid (catalog number: 37894) was obtained from Sigma-Aldrich. Stock solutions were prepared at 10 mg/mL dimethyl sulfoxide (DMSO; Sigma-Aldrich) and stored frozen at −80 °C until usage.

### *Drosophila melanogaster* husbandry

WT Canton-S (stock number: 1), *Rel* ^−/−^ (stock number: 55714), w^1118^ (stock number: 3605), *Upd1* ^−/−^ (stock number: 55728), *Upd2/3* ^−/−^ (stock number: 55729), FM7A (stock number: 785), *Hop2* ^−/−^ (stock number: 6032) and y^1^ w, ^*^P{UAS-D*pt*-cherry}C1 (*Dpt*-*RFP*, stock number: 55706) stocks were obtained from Bloomington Drosophila Stock Center at Indiana University. *D*. *melanogaster* were maintained using media containing 1.5% agar (w/v), 1.73% yeast (w/v), 7.3% cornmeal (w/v), 7.6% corn syrup (v/v), and 0.58% propionic acid (v/v) at 25 °C with 12 h light/dark cycles. For experimental procedures media were supplemented with or without pesticide prior to agar solidification. Unless otherwise stated, all experiments with adult *D*. *melanogaster* were performed using newly eclosed female flies. All experiments were performed in polypropylene *D*. *melanogaster* vials (GEN32-121 and GEN49-102, Diamed Lab Supplies Inc., Mississauga, ON, Canada) containing 10 ml of food media with 15–25 flies per vial.

### Bacterial strains and cultures

*Lactobacillus plantarum* (strain designation: Lp39 [IAM 12477]) was obtained from the American Type Collection Centre (ATCC number: 14917) and was cultured in MRS (de Man, Rogosa, and Sharpe [catalog number: 288130]; BD Difco). Lp39 used in experimental conditions were subcultures (1:100 dilutions) grown overnight (18 h) under anaerobic, stationary conditions at 37 °C. *Serratia marcescens* NCIMB 11782 was obtained from Lydia Dafoe (The University of Western Ontario) and was cultured in Luria-Bertani broth (LB, [catalog number: DF0446173]; BD Difco). *Serratia marcescens* Db11 was provided by the Caenorhabditis Genetics Center, which is funded by NIH Office of Research Infrastructure Programs (P40 OD010440) and the strain was cultured in LB broth containing 100 µg/mL streptomycin. *Serratia marcescens* strains used in experimental conditions were subcultures (1:100 dilutions) grown overnight under aerobic shaking (200 rpm) conditions at 37 °C.

### IMI-exposed *D*. *melanogaster* survival assays

Twenty-25 newly eclosed flies were anesthetized with CO_2_ and randomly transferred into standard vials containing IMI-supplemented (10, 50, and 100 µM doses) food or vehicle (DMSO) at mid-light cycle^56^. Following anesthetization, flies were confirmed to be alive, and subsequently monitored daily (9 AM) for survival. Flies were transferred to fresh media every 3 d.

### Culture-based *D*. *melanogaster* microbiota enumeration

*D*. *melanogaster* were surface sterilized with 70% ethanol and homogenized in 0.01 M PBS using a motorized pestle. Homogenates were then serially diluted and spot plated onto MRS and ACE (*Acetobacter* growth media containing 3 g/L proteose peptone no. 3 [catalog number: 211693; BD Difco], 5 g/L yeast extract [catalog number: 212750; BD Difco], and 25 g/L D-mannitol [catalog number: M9647; Signma-Aldrich]) agar, followed by anaerobic incubation at 37 °C for 48 h or aerobic incubation at 37 °C for 48 h to enumerate *Lactobacillus* spp. and *Acetobacter* spp., respectively. Colonies forming units on MRS and ACE were enumerated and confirmed to be *Lactobacillus* and *Acetobacter* spp., respectively, via microscopy analysis and 16S rRNA gene sequencing using the Applied Biosystems 3730 Analyzer platform at the London Regional Genomics Centre (Robart’s Research Institute, London, Canada).

### *D*. *melanogaster* eclosion assays

*Drosophila melanogaster* eggs were collected on grape agar plates^57^. For each vial ten first-instar larvae were transferred to *D*. *melanogaster* media with or without IMI (1 or 10 µM), incubated at the aforementioned conditions, and monitored daily for up to 16 d for eclosion.

### *D*. *melanogaster* heat stress assay

Newly eclosed female flies reared on media containing 10 μM IMI or vehicle were anesthetized with CO_2_ and randomly transferred into fresh vials containing the same media they were reared on previously. Following anesthetization, flies were confirmed to be alive and then subsequently exposed to lethal heat stress (37 °C) and monitored hourly for survival.

### Adult *D*. *melanogaster* septic infection

Newly eclosed (within 2–4 d) female flies that were reared from eggs on media containing vehicle or 10 μM IMI were infected by septic pinprick (approximately 100 bacteria per fly) as described previously^58^. Briefly, overnight cultures of *S*. *marcescens* NCIMB 11782 were washed with 0.01 M phosphate-buffered saline (PBS). Washed cultures were pelleted by centrifugation at 5000 g for 5 min, and supernatants discarded. Glass microinjection needles were made using a glass micropipette puller and sterilized with 70% ethanol before being used. Microinjection needles were dipped in the *S*. *marcescens* NCIMB 11782 bacterial pellet prior to pin pricking *D*. *melanogaster* at the sternopleural plate of the thorax just under the attachment sites of the wings. Alternatively, vehicle groups were subjected to septic injury via pinprick with a sterile needle. Following infection, groups of 10 flies were transferred to new tubes containing the same media they were raised on and monitored hourly for survival at 25 °C. Flies collected 0 h, 1 h, 3 h and 6 h post-infection were surface sterilized with 70% ethanol, homogenized, and plated on LB agar for pathogen load. Colony forming units were enumerated following 48 h incubation at 22 °C (*S*. *marcescens* NCIMB 11782 colonies appear red under these conditions).

### Larval *D*. *melanogaster* oral infections

Third-instar larvae, reared from eggs on media containing vehicle or 10 μM IMI, were orally infected with *S*. *marcescens* Db11 as described previously^60^. Larvae collected at 6 h, 12 h, and 24 h post-infection were surface sterilized with 70% ethanol, homogenized, and then plated on LB agar with 100 μg/mL streptomycin for overnight incubation at 37 °C to enumerate pathogen load.

### Lactobacilli-mediated IMI metabolism/binding assay

Overnight bacterial subcultures were pelleted at 5000 g for 10 min. Pellets were washed and re-suspended in 50 mM HEPES (pH 6.8). Bacterial-buffer or buffer-alone solutions were incubated with IMI (100 ppm) protected from light at 37 °C for 24 h (unless otherwise stated) with shaking (200 rpm). Cultures were pelleted and supernatant collected then analyzed for IMI levels using liquid-chromatography mass spectrometer consisting of an Agilent 1290 Infinity High Performance Liquid Chromatography (HPLC) coupled to a Q-Exactive Orbitrap mass spectrometer (Thermo Fisher Scientific, Waltham, USA) with a heated electrospray ionization (HESI) source. Two μL of each sample and standard were injected into a ZORBAX Eclipse plus C18 2.1 × 50 mm × 1.6 micron column. Mobile phase (A) consisted of 0.1% formic acid in water and mobile phase (B) consisted of 0.1% formic acid in acetonitrile. The initial composition of 100% (A) was held constant for 0.5 minutes and decreased linearly to 0% over 3.5 minutes. Mobile phase A was held at 0% for 1.5 minutes then returned to 100% over 30 seconds. The system was re-equilibrated at 100% for 1 minute, for a total analysis time of 6.50 minutes.

The HESI source was operated under the following conditions: nitrogen flow of 17 and 8 arbitrary units for the sheath and auxiliary gas, respectively. Probe temperature and capillary temperature were 450 °C and 400 °C, respectively. Spray voltage was 3.9 kV. The S-Lens was set to 45. A full MS and MS/MS scanning were used to monitor the parent mass and fragment ions respectively. A full MS scan between the ranges of 50–400 m/z in positive mode at 70,000 resolution (AGC target and maximum injection time were 3e6 and 250 ms respectively) was utilized. The MS/MS was set to 17,500 resolution and normalized collision energy set to 35 (AGC target and maximum injection time of 5 × 10^5^ and 65 ms, respectively).

### Bacterial imidacloprid tolerance assay

Overnight Lp39 cultures were sub-cultured (1:100 dilution) into 96 well plates (Falcon, catalog number: 351177) containing MRS broth with or without the addition of IMI or vehicle (DMSO). Plates were incubated at 37 °C and read every 30 min for 24 h at a wavelength of 600 nm using a Labsystems Multiskan Ascent microplate reader.

### Adult *D*. *melanogaster* septic infection mitigation assays with Lp39 supplementation

Overnight cultures were pelleted at 5000 g for 10 min, washed with 0.01 M PBS, and concentrated 10-fold with 0.01 M PBS. *Drosophila melanogaster* food was supplemented with 100 μL (10^9^ CFU) Lp39 when experimentally appropriate and allowed to air dry prior to usage. *Drosophila melanogaster* eggs were seeded on food media supplemented with Lp39 or vehicle (100 µL 0.01 M PBS) and reared until adult hood on this same media. Initial Lp39 supplementation occurred the day of egg seeding and additional supplementation followed every third day thereafter. Septic infections of supplemented flies were performed under the aforementioned experimental conditions.

### RNA extraction, reverse transcription, and qPCR

Adult female *D*. *melanogaster* were flash frozen using liquid nitrogen and stored at −80 °C until RNA extraction. Flies were homogenized in 500 µL of TRIzol reagent (Ambion, catalog number: 15596018) using an RNase-free pestle (Fisher Scientific, catalog number: 12-141-368) and total RNA was extracted according to manufacturer’s instructions with PureLink RNA Mini Kit (Ambion, catalog number: 12183018A). The quality of RNA was evaluated using a NanoDrop 2000 UV-Vis Spectrophotometer (Thermo Scientific) and determined to have A260/280 and A260/230 absorbance ratios between 2.0–2.3 and 1.9–2.4, respectively. cDNA was synthesized from 500 ng of total RNA using a High-Capacity cDNA Reverse Transcription Kit (Applied Biosystems, catalog number: 4368813).

Oligonucleotide primers were designed using Primer-BLAST software (https://www.ncbi.nlm.nih.gov/tools/primer-blast) and included: *Dpt* (NM_079063.4, 5′CCACTGGCATATGCTCCCAAT-CAAGGTGCTGGGCATACGAT3′, 190 bp), *TotA* (NM_080517.3, 5′GGTTTGCTTCAGCGTTCCAA-GCAGCAGTGCAAAGCACATAA3′, 75 bp), *RpLP0* (NM_079487.4, 5′CCGAAAAGTCTGTGCTTTGTTCT-CGCTGCCTTGTTCTCCCTAA3′, 83 bp), *GAPDH1* (NM_080369.3, 5′CCCAATGTCTCCGTTGTGGA-TGGGTGTCGCTGAAGAAGTC3′, 161 bp), *TBP* (NM_079081.4, 5′TCACAAAACGAGCCACAGGT-AGATTGCTGAACGGGCTCAA3′, 129 bp), *RpL13A* (NM_001275362.1, 5′TCTACAAGGCAGTCCGAGGT-AATAGCCGGTGAGTCAGGGA3′, 181 bp), and *ATUB84B* (NM_057424.4, 5′CAACTCCATCCTGACCACCC-GATCCACGTTAAGGGCACCA3′, 200 bp). Preliminary experiments identified *RpLP0* as being the most stably expressed reference gene (compared to *GAPDH1*, *TBP*, *RpL13A*, and *ATUB84B*) under experimental conditions in this study, and was thus used as the internal standard for normalization as per MIQE guidelines^61^.

cDNA was diluted 10-fold and used for qPCR reactions with the *Power* SYBR Green kit (Applied Biosystems, catalog number: 4368702). Reagent volumes for 20 µL reactions consisted of 10 µL *Power* SYBR (2x), 0.4 µL forward primer (10 µM stock), 0.4 µL reverse primer (10 µM stock), 4.2 µL nuclease-free H_2_O, and 5 µL cDNA. Reaction conditions were 95 °C for 10 min followed by 40 cycles of 95 °C for 15 sec and 60 °C for 1 min. qPCR was performed on a 7900HT Sequence Detection System (Applied Biosystems) and analyzed using SDS RQ 6.3 manager software (Applied Biosystems) and relative gene expression was calculated using the 2^−ΔΔct^ method^62^. PCR amplification was confirmed via melt-curve dissociation analyses to verify product size and check for non-specific amplification.

### Fluorescent microplate reader

Newly eclosed *Dpt*-RFP *D*. *melanogaster* were exposed to cotton gauze soaked in 2 mL 5% sucrose containing 10^9^ CFU of Lp39 or vehicle for 15 h at 25 °C. WT Canton-S flies exposed to identical experimental conditions were used as negative controls. Samples containing 10 flies were homogenized in 400 µL of 0.01 M PBS on ice using a BioSpec 3110BX Mini Beadbeater (Fisher Scientific, catalog number: NC0251414) with silica beads. Homogenates were centrifuged at 12,000 g for 20 minutes at 4° C. Supernatants (200 µL/sample) were added to Corning 96-well solid black polystyrene microplates for *Dpt* expression of the red fluorescent protein (RFP) reporter, mCherry. Plates were measured at an excitation wavelength/bandwidth of 587/9 nm and emission wavelength/bandwidth of 645/20 nm^63^ using a BioTek Synergy2 microplate reader (Fisher Scientific, catalog number: 36-101-5201).

### Confocal microscopy

Newly eclosed *Dpt*-RFP *D*. *melanogaster* were exposed to cotton gauze soaked in 2 mL 5% sucrose containing 10^9^ CFU Lp39 or vehicle for 15 h at 25 °C. *Dpt*-RFP *D*. *melanogaster* midguts were dissected and counterstained with 162 µM Hoechst 33342, trihydrochloride, tetrahydrate (Molecular Probes, catalog number: H3570). Using 4x and 20x objective lenses, the 4′,6-diamidino-2-phenylindole, dihydrochloride (DAPI, 408 nm) channel was utilized to provide background contrast and the Alexa594 (594 nm) channel was used to visualize *Dpt*-RFP expression. Multichannel and z-stack images were produced using NIS-Elements Advanced software (Nikon Inc., Tokyo, Japan).

### Statistical analyses

All statistics were performed using GraphPad Prism 7.0 software. Data sets with unique values were tested for normality using the omnibus-based Shapiro-Wilk test, while data set with ties (two or more identical values) were tested for normality using the D’Agostino-Pearson test. Normally distributed data were statistically compared with unpaired, two-tailed t tests, one-way or analysis of variance (ANOVA) or two-way ANOVA as indicated. ANOVA tests were complemented with Tukey’s (for data with one categorical variable) or Sidak’s (for data with two categorical variables) multiple comparison tests when appropriate. Nonparametric data sets were statistically compared using Mann-Whitney and Kruskal-Wallis tests (with Dunn’s multiple comparisons) when appropriate. Mantel-Cox tests were used to analyze overall *D*. *melanogaster* survival. Alternatively, Gehan-Breslow-Wilcoxon tests were used to analyze *D*. *melanogaster* survival with emphasis on early time point events. Multiple comparisons for Mantel-Cox and Gehan-Breslow-Wilcoxon tests were performed using the Bonferroni method.

## Electronic supplementary material

Supplementary Info
