# Supplementary material for: Neonicotinoid-induced pathogen susceptibility is mitigated by Lactobacillus plantarum immune stimulation in a Drosophila melanogaster model
Source: Sci Rep. 2017 Jun 2;7:2703. doi: 10.1038/s41598-017-02806-w (PMC5457429; doi:10.1038/s41598-017-02806-w)
Supplement: Supplementary file 1 — Supplementary Info [file 41598_2017_2806_MOESM1_ESM.pdf]

Neonicotinoid-induced pathogen susceptibility is mitigated by *Lactobacillus plantarum* immune stimulation in a *Drosophila melanogaster* model.

Brendan A. Daisley[1,2*], Mark Trinder[1,2*], Tim W. McDowell[3], Hylke Welle[1,2,4], Josh S. Dube[2], Sohrab N. Ali[2,5], Hon S. Leong[2,6], Mark W. Sumarah[3], and Gregor Reid[1,2,6,#].

[1]Centre for Human Microbiome and Probiotic Research, Lawson Health Research Institute, London, N6C 2R5, Canada. [2]Department of Microbiology and Immunology, The University of Western Ontario, London, N6A 5C1, Canada. [3]London Research and Development Center, Agriculture and Agri-Food Canada, London, N5V 3V3, Canada. [4]Vrije Universiteit Amsterdam, Faculty Earth and Life Sciences, Institute of Molecular Cell Biology, Amsterdam, 1081, Netherlands.[5]Department of Surgery, Division of Urology, University of Ottawa, Ottawa, K1Y 4E9, Canada. [6]Department of Surgery, The University of Western Ontario, London, N6A 4V2, Canada.

[#] Address correspondence to Dr. Gregor Reid, gregor@uwo.ca.

[*] These authors contributed equally to this work.

SUPPLEMENTARY METHODS

Rel**$^{-/-}$ **D. melanogaster **survival assays.** Twenty to twenty-five newly eclosed *Rel*$^{-/-}$ flies were anesthetized with CO_2 and randomly transferred into standard vials at mid-light cycle for each replicate[1]. Following anesthetization, flies were confirmed to be alive and then subsequently monitored daily (9AM) for survival. *Drosophila melanogaster* food media contained vehicle or varying concentrations of IMI (10, 50, and 100 µM doses). Surviving flies were transferred to fresh media every 3 d.

Dpt **expression during 10 µM IMI exposure.** *Dpt-RFP D. melanogaster* were reared on food containing 10 µM IMI or vehicle (DMSO). Briefly, samples containing 10 newly eclosed *Dpt*-RFP flies in 400µL PBS on ice were homogenized with silica beads using a BioSpec 3110BX Mini Beadbeater (Fisher Scientific, catalog number: NC0251414). Homogenate was then centrifuged at 12,000 g for 20 minutes in 4°C and resulting supernatant was used for subsequent analyses. *Dpt* expression was determined by measuring relative fluorescence intensity of the red fluorescent protein (RFP) reporter, mCherry. Fluorescence intensity was recorded using an excitation wavelength/bandwidth of 587/9nm and emission wavelength/bandwidth of 645/20nm[2] using a BioTek Synergy2 microplate reader (Fisher Scientific, catalog number: 36-101-5201).

Abx-treated *D.melanogaster* survival assays. Twenty-five newly eclosed *Rel*$^{-/-}$ flies were anesthetized with CO_2 and randomly transferred into standard vials containing 100 µM IMI or vehicle (DMSO) with or without antibiotics[3] at mid-light cycle for each replicate[1]. Following anesthetization, flies were confirmed to be alive and then subsequently monitored daily (9AM) for survival. *Drosophila* food media contained vehicle or varying concentrations of IMI (10, 50, and 100 µM doses). Surviving flies were transferred to fresh media every 3 d.

SUPPLEMENTARY REFERENCES

1. Linford, N. J., Bilgir, C., Ro, J. & Pletcher, S. D. Measurement of lifespan in *Drosophila melanogaster*. *JoVE J. Vis. Exp.* e50068–e50068 (2013). doi:10.3791/50068.
2. Duellman, T., Burnett, J. & Yang, J. Quantitation of secreted proteins using mCherry fusion constructs and a fluorescent microplate reader. *Anal. Biochem.* **473,** 34–40 (2015).
3. Brummel, T., Ching, A., Seroude, L., Simon, A. F. & Benzer, S. *Drosophila* lifespan enhancement by exogenous bacteria. *Proc. Natl. Acad. Sci. U. S. A.* **101,** 12974–12979 (2004).

SUPPLEMENTARY FIGURES

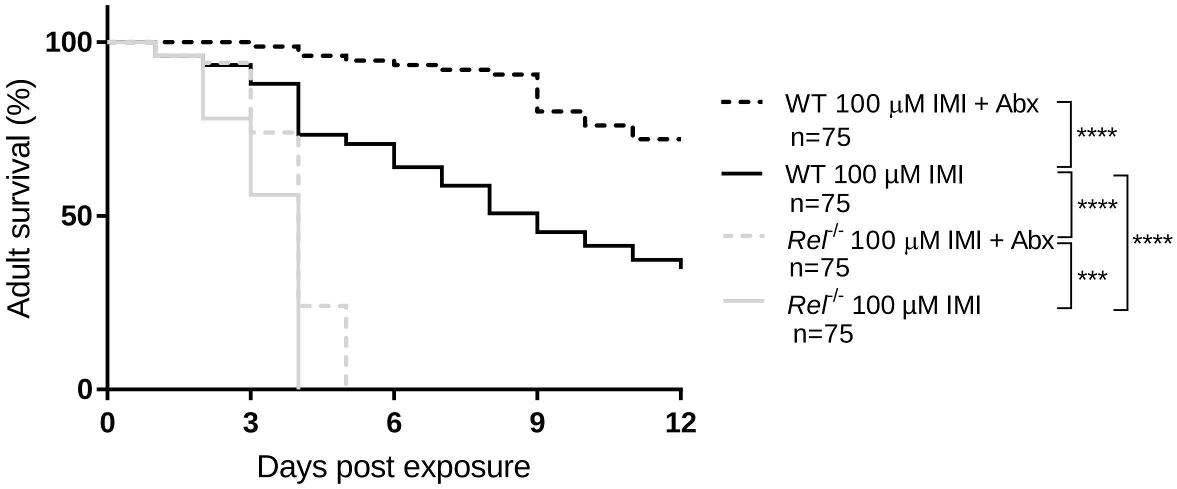

Supplementary Figure 1. Abx-treated WT and *Rel*$^{-/-}$ *D. melanogaster* are less susceptible to IMI toxicity. Survival curves for newly eclosed WT and *Rel*$^{-/-}$ flies fed food containing vehicle or food containing 100 µM IMI with or without antibiotics. All statistical symbols are representative of comparisons made using the log-rank (Mantel-Cox) test. Data are displayed from at least 3 independent experiments (25 flies each group per experiment). **** p<0.0001, *** p<0.001.

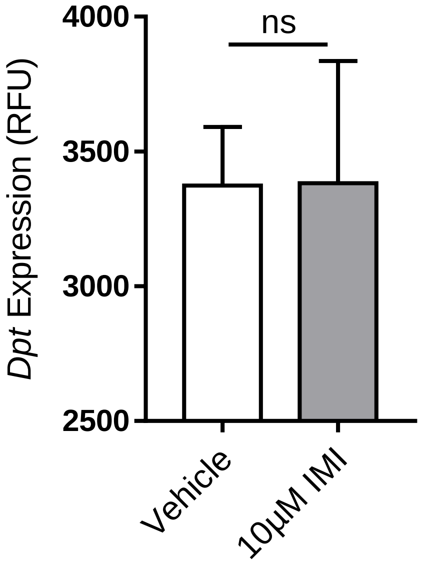

Supplementary Figure 2. *Dpt* expression in response to IMI exposure. *Dpt* expression of newly eclosed *Dpt*-RFP reporter flies fed food containing 10 µM IMI and vehicle (DMSO) was determined. No significant difference (unpaired, two-tailed t-test; t = 0.04937, df = 14, *P* = 0.9613) in *Dpt* expression was noted between vehicle and 10 µM IMI-exposed *Dpt*-RFP report flies. Gene expression was quantified by fluorescence intensity using a microplate reader. Mean ± standard deviations of 8 independent biological replicates (10 flies per replicate) for each group is shown. RFU = Relative Fluorescent Units. ns = not significant

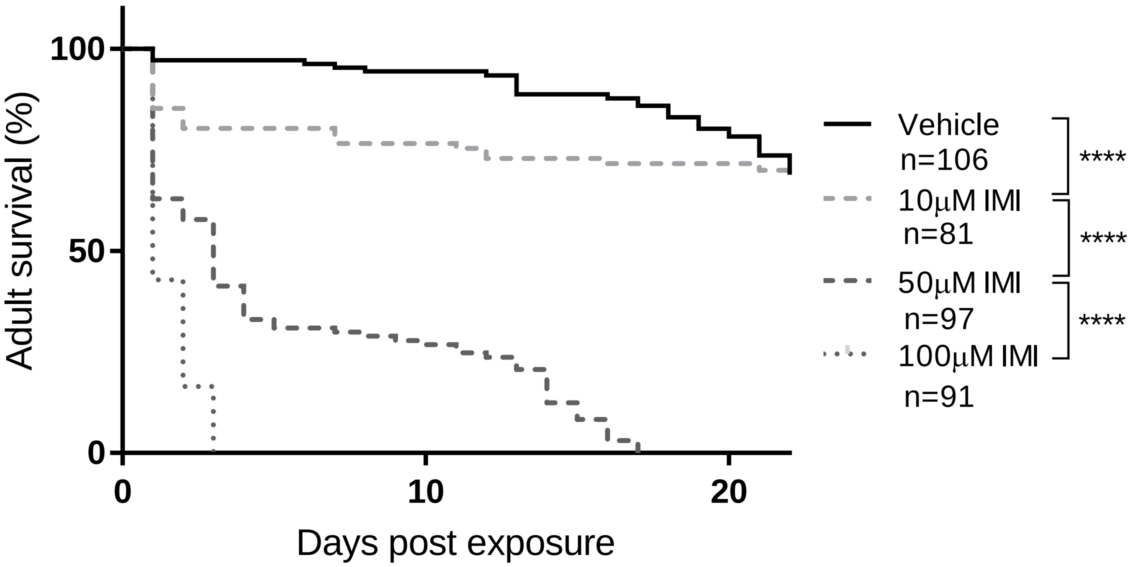

Supplementary Figure 3. IMI exposure in *Rel*$^{-/-}$ *D. melanogaster* results in dose dependent toxicity. Survival curves for newly eclosed *Rel*$^{-/-}$ flies fed food containing vehicle (DMSO) or food containing varying concentrations of IMI (10, 50, and 100 µM). Data are displayed from at least 3 independent experiments (20-25 flies each group per experiment). All statistical symbols are representative of comparisons made using the log-rank (Mantel-Cox) test. **** p<0.0001.
